# BRANEnet: embedding multilayer networks for omics data integration

**DOI:** 10.1186/s12859-022-04955-w

**Published:** 2022-10-17

**Authors:** Surabhi Jagtap, Aurélie Pirayre, Frédérique Bidard, Laurent Duval, Fragkiskos D. Malliaros

**Affiliations:** 1 Université Paris-Saclay, CentraleSupélec, Inria, 3 Rue Joliot Curie, 91190 Gif-Sur-Yvette, France; 2 IFP Energies nouvelles, 1 et 4 avenue de Bois-Préau, 92852 Rueil-Malmaison, France

**Keywords:** Multi-omics data, Multilayer network, Biological network integration, Graph representation learning, Regulatory network inference

## Abstract

**Background:**

Gene expression is regulated at different molecular levels, including chromatin accessibility, transcription, RNA maturation, and transport. These regulatory mechanisms have strong connections with cellular metabolism. In order to study the cellular system and its functioning, omics data at each molecular level can be generated and efficiently integrated. Here, we propose BRANEnet, a novel multi-omics integration framework for multilayer heterogeneous networks. BRANEnet is an expressive, scalable, and versatile method to learn node embeddings, leveraging random walk information within a matrix factorization framework. Our goal is to efficiently integrate multi-omics data to study different regulatory aspects of multilayered processes that occur in organisms. We evaluate our framework using multi-omics data of *Saccharomyces cerevisiae*, a well-studied yeast model organism.

**Results:**

We test BRANEnet on transcriptomics (RNA-seq) and targeted metabolomics (NMR) data for wild-type yeast strain during a heat-shock time course of 0, 20, and 120 min. Our framework learns features for differentially expressed bio-molecules showing heat stress response. We demonstrate the applicability of the learned features for targeted omics inference tasks: transcription factor (TF)-target prediction, integrated omics network (ION) inference, and module identification. The performance of BRANEnet is compared to existing network integration methods. Our model outperforms baseline methods by achieving high prediction scores for a variety of downstream tasks.

**Supplementary Information:**

The online version contains supplementary material available at 10.1186/s12859-022-04955-w.

## Background

Gene expression in eukaryotes is regulated at different molecular levels, including chromatin accessibility, transcription, RNA maturation, and transport. These regulatory processes are affected by specific bio-molecules potentially in separate cellular compartments with interconnected steps [1]. One of the earlier steps consists of epigenetic modifications that can modify DNA accessibility and chromatin structure, ensuring access to the transcriptional machinery [2, 3]. These modifications occur across the genome, regulating the final synthesis of the mRNAs [4]. These newly synthesized transcripts (mRNAs) are extensively modified and translated into proteins [5]. In this process, mRNA molecules are guided by RNA-binding proteins that control their expression [6]. Finally, the encoded protein products (metabolites) participate in numerous processes that regulate cellular metabolism [7, 8]. The metabolites produced in this step are transformed and/or stored. A number of these metabolites/compounds (for instance, Acetyl-CoA, glucose or methyl groups) successively participate in chromatin modifications (epigenetics) that regulate gene expression [9, 10]. A comprehensive interpretation of such phenomena would help to understand the structure, function, and dynamics of cellular systems [11]. For this purpose, the collective characterization and quantification of data at different molecular levels are essential. Omics technologies give access to these regulatory mechanisms and are thus able to provide large amounts of data at each molecular level. Despite the abundance and diversity of numerous available omics datasets, there are some noticeable challenges regarding their acquisition, processing, efficient integration, and interpretation [11–13].

To this end, networks are widely used to represent individual bio-molecules (nodes) and their biological relationships (edges). These relationships may reflect gene co-expression/regulation, protein–protein interactions (PPIs) or information about production or consumption of metabolites [14]. The major challenge thus pertains to effectively integrate the variety of these relationships (layers) encoded in multiple networks. In this study, we are inspired by graph representation learning (GRL) algorithms that allow to encode the high-dimensional graph structure into compact low dimensional embedding vectors [15, 16]. The aim is to optimize the mapping from the original graph-based node representation onto a lower dimensional space such that their geometric relationship in this latent space reflects the structure of the input graph (network). After optimizing the embedding space, the learned embeddings can be used as input features for downstream omics inference tasks, such as bio-marker identification, drug-target prediction, disease-gene associations, gene regulatory network (GRN) inference [17], and protein function prediction [13].

In the related literature, a variety of methods have been proposed for computing network embeddings either based on random walks [18, 19], matrix factorization [20], or neural networks [15]. However, the majority of them has specifically been designed for single-layer networks. Nevertheless, for multilayer biological networks, only a few existing network integration strategies leverage GRL. As one of the proposed models, OhmNet [21] is a hierarchy-aware task-independent, unsupervised method for protein feature learning. Multi-Net [22] is an extension of the DeepWalk [18] and Node2Vec [19] to multilayer graphs. It allows to perform random walks by defining paths to traverse the nodes. deepNF [23], a network fusion method, is based on multimodal deep autoencoder (MDA) to integrate different heterogeneous networks. BRANE-Exp [24] leverages random walks with the concept of exponential family graph embeddings [25, 26]. It generalizes multilayer random walk-based GRL methods to an instance of exponential family distributions. More recently, MOSS [27] performs a sparse singular value decomposition (sSVD) to learn embeddings. Nevertheless, most of the existing methods are challenged when applied to real and heterogeneous omics data which demands comprehensive handling towards preserving biological relevance [11]. In such cases, it is necessary to obtain knowledge-based representations of the nodes to assist omics data analysis. Here, we propose BRANEnet, a novel multi-omics integration framework for multilayer heterogeneous networks. In particular, we embed graph-based information from multi-omics data into a lower-dimensional space. We leverage a properly chosen random walk-based Positive Pointwise Mutual Information (PPMI) matrix, that is able to capture relevant context around each node of interest. We introduce network integration with multilayered heterogeneous graph embeddings, that perform matrix factorization by approximating the spectrum of this PPMI matrix. We apply BRANEnet over a multi-omics dataset for heat-shock response in the well-known yeast model *Saccharomyces cerevisiae* [28] and learn embeddings for genes, transcription factors (TFs), and metabolites. We focus on TF-target prediction, integrated omics network (ION) inference, and module detection. We validate the embeddings on various downstream tasks and demonstrate BRANEnet’s effectiveness by comparing its performance to existing network integration approaches.

## Methods

### Data description

**Fig. 1:**
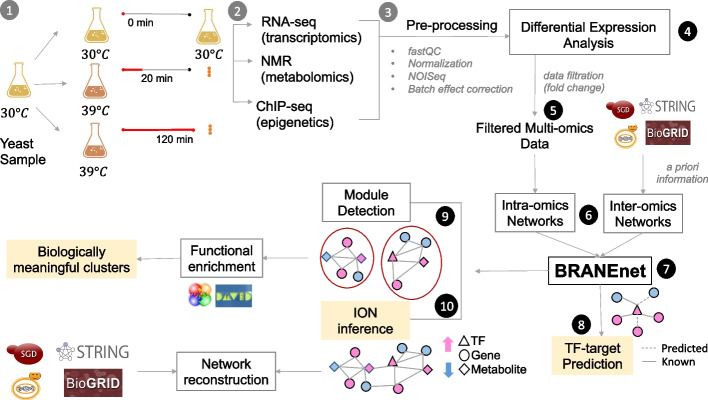
Experimental design and BRANEnet processing workflow. The set up of wet-lab experiments (steps 1, 2, and 3) are taken from the data descriptor article [28]. Steps 4, 5, and 6 perform dataset collection and prepossessing before integration. (7) Learn embeddings using BRANEnet. (8–10) Downstream bioinformatics tasks

To test our framework, we have used recently published multi-omics yeast datasets acquired in the study of [28]. These datasets are obtained with the same yeast sample presenting three basic layers of the transcriptional circuit, including: one type of epigenetic modification (H4K12ac mark for identification of active promoters obtained from ChIP-Seq), gene expression (RNA-seq), and targeted metabolomics (NMR). The dataset is comprised of 7126 genes, 1970 H4K12ac peaks, and 37 metabolites. To obtain this data, yeast culture flask was grown at $30\,^\circ$C until the exponential phase. This culture was split into three different flasks. One flask was maintained at $30\,^\circ$C and labeled as 0 minute (t0). The other two flasks were incubated at $39\,^\circ$C for 20 min (t20) and 120 min (t120), respectively. Aliquots from all three flasks (t0, t20, and t120) were collected for ChIP-seq (epigenomics), RNA-seq (transriptomics), and NMR (metabolomics). This process was repeated four times to generate four biological replicates. The datasets were pre-processed using various bioinformatics tools [28]. These consistent datasets, dedicated to the study of the heat stress response, appear as a good candidate to test and evaluate our proposed omics data integration methodology. We recall the experimental setup and summarize the workflow in Fig. 1.

### Differential expression analysis

Genes, metabolites, TFs are hereafter referred to as bio-molecules. More generally, genes are regulated by TFs. Therefore, we separate TFs (genes coding for TFs) and non-TFs (genes not coding for TFs) from transcriptomics data. Now, to obtain differentially expressed bio-molecules, we first take the average of control samples in the four biological replicates. Then, we compute the log2 of Fold Change (log2FC) for each bio-molecule in eight test samples (four in t20 and four in t120) by taking its ratio against the average of four control samples (t0). For each test sample, we select non-TFs if log2FC is higher than 2 (over-expressed) or lower than $-2$ (under-expressed). However, it is well known that expression of TFs do not vary considerably as compared to non-TFs [29]. Therefore, we lower the threshold of log2FC for genes encoding for TFs. TFs were considered as differentially expressed, if log2FC is higher than 1 (over-expressed) or lower than $-1$ (under-expressed). For metabolites and H4K12ac peaks, we choose the log2FC threshold similarly to TFs. If a log2FC value is meaningful with respect to the above thresholds in at least one biological replicate, we consider the corresponding bio-molecule as differentially expressed.

### Construction of intra-omics and inter-omics networks

Intra-omics networks are constructed using the same type of bio-molecules, for example, gene-gene co-expression, or metabolite-metabolite correlation networks. These networks are built on data obtained from multi-omics experiments, for instance: genomics, epigenetics, transcriptomics, proteomics, and metabolomics. We obtain differentially co-expressed bio-molecules by computing the Pairwise Pearson correlation coefficient ($\rho$) [30] of log2FC profiles described above, i.e., log2FC for the eight samples (four in t20 and four in t120). Pair-wise intra-omic correlations for which the absolute value of $\rho$ is higher than 0.8, were obtained. These intra-omic correlation networks are represented as a set of their adjacency matrices.

Inter-omics networks link bio-molecules of different types. They are constructed using biological a priori information showing presence of TF binding sites or H4K12ac epigenetic marks in the promoter of gene, biochemical reactions within genes and metabolites. This information can be acquired from various bioinformatics databases such as SGD [31], YEASTRACT (Yeast Search for Transcriptional Regulators And Consensus Tracking) [32]), YeastPathways [31], and BioCyc [33]. This a priori knowledge bridges the gap to relate two different omics types, for instance gene-metabolite, TF-target, gene-epigenetic mark. For each differentially expressed bio-molecule of one type (e.g., gene), we obtained its relationship with an bio-molecule of another type (e.g., TF and metabolite).

### The BRANEnet model

**Fig. 2:**
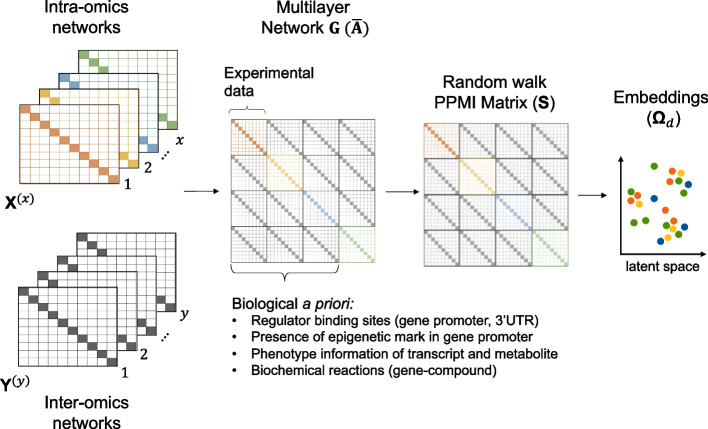
Overview of BRANEnet. Inputs (*X*) and (*Y*) are on the left. A multilayer network ${ \bar{\mathbf {A}}}$ is composed of intra- and inter-omics relationships. For ${\bar{\mathbf {A}}}$, the random walk-based PPMI matrix ${\mathbf {S}}$ is computed. To obtain embeddings, ${\mathbf {S}}$ is factorized and the final embeddings $\varvec{\Omega }_d$ are obtained

In Fig. 2, an illustration of BRANEnet is presented. The model takes two sets of networks, intra- and inter-omics *X* and *Y* respectively. It first builds a multilayer network *G* using both sets which are represented by ${\bar{\mathbf {A}}}_{|N| \times |N|}$, where *N* is a set of all bio-molecules. Then, we compute a random walk-based PPMI matrix ${\mathbf {S}}$ for ${\bar{\mathbf {A}}}$ via a closed-form solution. Matrix ${\mathbf {S}}_{|N| \times |N|}$ is then factorized using Singular Value Decomposition (SVD), and the *d*-dimensional embedding vectors $\varvec{\Omega }_d \in {\mathbb {R}}^{|N| \times d}$ ($d \ll |N|$) are given by ${\mathbf {U}}_d \sqrt{\varvec{\Sigma }_d}$. Next, we describe the workflow in detail.

#### Construction of a multilayer network

*X* is a set of *x* intra-omics networks represented as ${\mathbf {X}}^{(1)}_{n_1 \times n_1}, {\mathbf {X}}^{(2)}_{n_2 \times n_2}, \ldots , {\mathbf {X}}^{(x)}_{n_x \times n_x}$, while *Y* is a set of $y = \frac{x(x-1)}{2}$ inter-omics networks represented as ${\mathbf {Y}}^{(1)}_{n_1 \times n_2}, {\mathbf {Y}}^{(2)}_{n_1 \times n_3}, \ldots , {\mathbf {Y}}^{(y)}_{n_{x-1} \times n_x}$. A multilayer network *G* of |*N*| nodes (bio-molecules) and |*E*| edges (interactions) is built using sets *X* and *Y*. The network is represented by its supra-adjacency matrix ${\bar{\mathbf {A}}}_{|N| \times |N|}$ that is defined as:

1 $$\begin{aligned} {\bar{\mathbf{A}}}= \bigoplus _x {\mathbf {A}}^{(x)} + {\mathbf {C}}, \end{aligned}$$

where $\bigoplus _x{\mathbf {A}}^{(x)}$ is the intra-omics adjacency matrices and ${\mathbf {C}}$ is a block matrix with zero diagonal blocks that stores inter-omics connections obtained from elements in *Y*. The final output of ${\bar{\mathbf{A}}}$ has intra-omics networks represented as blocks in the main diagonal and inter-omics networks represented as off-diagonal matrices.

#### Representation learning

To embed nodes from different omics modalities into a common latent space towards integrating inter- and intra-omics relationships, we construct a random walk matrix ${\mathbf {S}}$ for the multilayer graph *G*. ${\mathbf {S}}$ is defined by the random walk transition probabilities to traverse nodes within and across layers. The flexibility of random walks to traverse within and across layers allows us to capture inter- and intra-layer node neighbourhood information. This is an important and useful property to consider while performing data integration from multilayer networks [22, 24]. For instance, starting from node *v* in *G*, a random walk traverses the multilayer graph, moving across neighborhood nodes of *v* chosen uniformly at random. This process repeats for a predefined number of walks per node. Nevertheless, for large networks, simulating random walks is computationally expensive. In particular, the space and time complexity for DeepWalk is ${\mathcal {O}}(|N|^2)$ and ${\mathcal {O}} (\gamma lT|N|(d + d$log|*N*|)), respectively. Here, |*N*| denotes the number of nodes; $\gamma$ is the number of walks; *l* is the walk length; and *T* denotes the window size [34]. To address this limitation, we leverage the relationship between random walk-based GRL algorithms that rely on the Skip-Gram model (for instance, DeepWalk [18]) and matrix factorization [35]. In particular, a multilayer random walk matrix ${\mathbf {S}}$ is defined by computing the closed-form of a properly normalized PPMI based random walk transition matrix. This PPMI matrix is the well-studied pointwise mutual information (PMI) matrix that represents node similarities, shifted by a global constant. It has been shown that normalized PPMI is better at optimizing Skip-Gram’s objective and shows better performance than word2vec derived models [18, 19] in Natural Language Processing (NLP tasks) [35, 36]. The space and time complexity is now ${\mathcal {O}}(|E| +k|N|)$ and ${\mathcal {O}}(qk(|E| +k|N|))$ [37], where |*E*| is the number of edges; *q* denotes the power parameter; and *k* is the rank parameter. For any graph *G*, ${\mathbf {S}}$ is given by:

2 $$\begin{aligned} \begin{aligned} {\mathbf {S}} = \log \left\{ \frac{{\text {vol}}(G)}{bT}\left( \frac{1}{T}\sum _{r=1}^{T}{\mathbf {P}}^{r}\right) {\mathbf {D}}^{-1}\right\} , \end{aligned} \end{aligned}$$

where ${\mathbf {P}} = {\mathbf {D}}^{-1} \bar{\mathbf {A}}$. Matrices $\bar{\mathbf {A}}$ and ${\mathbf {D}}$ are respectively the adjacency and diagonal degree matrices of the graph *G* and ${\text {vol}}(G)$ is the sum of the node degrees of *G*. *T* corresponds to the window size and *b* is number of negative samples [35]. In order to obtain node embeddings from the matrix ${\mathbf {S}}$, we perform spectral decomposition using SVD [38], given by ${\mathbf {S}} = {\mathbf {U}} \varvec{\Sigma } {\mathbf {V}}^\top$. Since ${\mathbf {S}}$ is a real and symmetric matrix, ${\mathbf {U}}$ and ${\mathbf {V}}$ correspond to singular vector matrices and $\varvec{\Sigma }$ is the singular value matrix. The integrated embedding matrix $\varvec{\Omega }_d$ of dimension $|N| \times d$ is given by the first *d* eigenvectors of ${\mathbf {S}}$, appropriately weighted by the square root of $\varvec{\Sigma }_d$ as $\varvec{\Omega }_d = {\mathbf {U}}_d\sqrt{\varvec{\Sigma }_d}$.

### Downstream tasks

The embeddings learned from BRANEnet can be used for various downstream tasks, for instance TF-target prediction, ION inference, identification of biomarkers (e.g. heat stress responsive genes/TFs), identification of biologically related clusters, and visualization. Their details are shown below.

#### TF-target prediction

To predict TF-targets, we adapt the traditional link prediction task [39] to TF-target networks. We use the largest connected component of the TF-target network. Then, we split the targets of each TF into two parts to form positive training and test sets by randomly removing $50\%$ of them. The same number of TF-target pairs that do not exist are sampled to generate negative instances for each training and test sets. The learned embeddings $\varvec{\Omega }_d$ are used to compute edge features. In particular, the embeddings of node *i* and *j* of size *d*, given by $\varvec{\Omega }_d[i]$ and $\varvec{\Omega }_d[j]$ respectively, are converted into edge feature vectors using element-wise operations [19, 26] (i) *average*: $(\varvec{\Omega }_d[i] + \varvec{\Omega }_d[j]) / 2$; (ii) *weighted* L2: $|(\varvec{\Omega }_d[i] - \varvec{\Omega }_d[j])|^2$. Now, for each positive and negative test and training dataset generated above, edge features are computed. Then, we perform prediction using the logistic regression classifier with L2 regularization [40]. The performance is measured using the area under the precision-recall curve (AUPR) [41]. The performance of BRANEnet for TF-target prediction is compared with baseline methods.

#### ION inference

To infer an ION from the learned embeddings, the pairwise similarity score for nodes *i* and *j* is defined as:

3 $$\begin{aligned} \delta _{i,j} = \varvec{\Omega }_d[i] \cdot \; \varvec{\Omega }_d[j] = \displaystyle \sum _{k=1}^{d} \varvec{\Omega }_d[i]_{k} \varvec{\Omega }_d[j]_{k}. \end{aligned}$$

To validate this network, we compare it with the gold-standard (GS) network of yeast that is built by combining networks from multiple databases, such as BIOGRID [42], STRING [43], and YEASTRACT [44]. The performance of ION inference is measured by computing the Precision@*k* and the Matthews Correlation Coefficient (MCC). Precision@*k* gives us the fraction of true edges among the inferred top *k* ones. To compute Precision@*k*, we sort edges based on the pairwise similarity score, then take the top *k* ones. Then, we compute Precision ($\frac{{\text {True}}\,{\text{ Positive}}\, ({\text{TP}})}{{\text {TP}} + {\text {False}}\,{\text{Positive}}\, ({\text{FP}})}$) at each top *k* edges. We chose $k \in \{1,10,50,100,\ldots , 500\}$. MCC is another way to evaluate performance. It measures the differences between the actual values and the predicted ones. The advantages of MCC over Precision-based metrics are shown in recent article [45]. For the edges with similarity score $\delta _{i,j}$ higher than a threshold ($\theta \in \{0.1,0.2, \ldots ,0.9\}$), we compute MCC ($\frac{{\text { True}}\,{\text{Negative}}\, ({\text{TN}}) \times {\text {TP}} - {\text {False}}\,{\text{ Negative}}\, ({\text{FN}}) \times {\text {FP}}}{\sqrt{({\text {TP}} + {\text {FP}})\, ({\text {TP}} + {\text {FN}}) \,({\text {TN}} + {\text {FP}})\, ({\text {TN}} + {\text {FN}}))}}$). We compare the performance of BRANEnet for ION inference with baseline methods.

#### Module detection

Interestingly in biological networks, the clustering or community structure property is present, under which the graph topology is organized into modules commonly called communities or clusters. To obtain these modules, we first select the top scoring edges ($\theta = 0.7$). Then we find clusters using a greedy modularity maximization algorithm [46]. We select the obtained modules having more than 10 nodes. To validate the obtained clusters, we have investigated their biological meaningfulness by performing functional annotation enrichment analysis [47, 48].

### Comparison with baseline methods

We compare the performance of link prediction and ION inference using the embeddings learned by BRANEnet with existing multilayer network embedding methods. We choose OhmNet [21], MultiNet [22], deepNF [49], BRANE-Exp, [24] and MOSS [27] as our baseline methods. These network integration methods are not specifically developed for multi-omics integration considering biological a priori knowledge to learn node embeddings. Therefore, we adapt the existing methods for learning embeddings and performing downstream tasks by keeping the same empirical parameter settings as of BRANEnet. Apart from deepNF, all baseline models mainly depend on the window size (*T*) and embedding dimension (*d*). For deepNF, we choose to keep the default model architecture configuration proposed by the authors [49].

## Results and discussion

We present the results of BRANEnet applied to the yeast multi-omics dataset [28]. We have identified differentially expressed (DE) bio-molecules as mentioned in the “Methods” section. We have obtained 333 DE genes (non-TF) out of which 310 are upregulated and 23 are downregulated, 55 DE TFs (50: over-expressed; 5: under-expressed), 30 DE metabolites (28: increased concentration; 2: decreased concentration). The list of all DE bio-molecules with their FC and SGD annotation is provided in the Additional file 1. For the epigenetics data, we have observed that no H4K12ac peaks were differentially expressed. Therefore, we discard ChIP-Seq data and use only variable genes, TFs, and metabolites for the study of heat shock response. We then compute intra- and inter-omics networks as described in the “Construction of a multilayer network” section. To construct inter-omics relationships, we obtain known TF-target interactions from the YEASTRACT database [32]. Gene-metabolite and TF-metabolite associations were given by the participation of genes or TFs in production and consumption of metabolites in biochemical reactions. This information was acquired from the YeastPathways database [31]. Embeddings are then learned for each node, as discussed in the “Methods” section. We use these embeddings to study different aspects of multi-omics data integration, namely TF-target Prediction, ION inference, and module detection.

### TF-target prediction

**Fig. 3:**
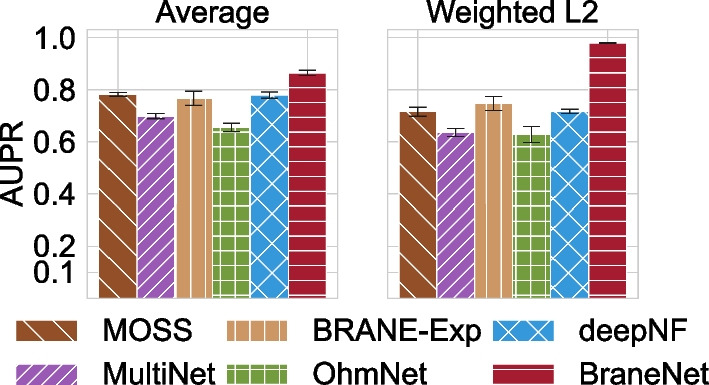
TF-target prediction. Comparative performance of BRANEnet with baseline methods with AUPR scores for both *average* and *weighted L2* coordinate-wise operations. The error bars show the standard deviation in AUPR scores for 10 runs

We have performed TF-target prediction as mentioned in the “Methods” section. First, we compute node embeddings using BRANEnet with parameter $T=3$, $b=1$, and $d=128$. For the same value of *d*, we learn node embeddings using each baseline method. The edge features are computed using the operators mentioned in the “Methods” section. TF-target prediction is then performed using logistic regression and its performance is measured using the AUPR score. Since, we randomly remove $50\%$ of targets for each TF, we repeat this process 10 times and report the average AUPR scores with standard deviation computed across 10 runs. The results for BRANEnet compared to baselines models are summarized in Fig. 3. The average AUPR of BRANEnet is $~10\%$ improved compared to the *average* ($87\%$) and to the *weighted L2* ($97.9 \%$) operators. For the empirical parameter settings, the performance of BRANEnet is higher in both operators as compared to the baselines methods. *Weighted L2* score of BRANEnet’s is $20\%$ higher than BRANE-Exp, the second best performing model. Whereas BRANEnet’s *average* score is $10\%$ higher then MOSS, the second best performing model for *average*. The standard deviation of BRANEnet for 10 runs is notably lower (except MOSS with *average*) than all the other methods. Overall from Fig. 3, we observe that BRANEnet outperforms the baseline methods for both operators (i.e., *average* and *weighted L2*). With the exception of deepNF, all the other algorithms constitute mostly extensions of Skip-Gram-like GRL models (e.g., DeepWalk) to multilayer graphs. For single layer graphs, the computational advantages of the spectral SVD algorithm over stochastic gradient training have been well-studied [36, 50]. First, it is exact, therefore does not require extensive hyper-parameter tuning. Second, unlike Skip-Gram’s training procedure, BRANEnet does not require layer-wise observations of each node and its respective neighborhood. Hence, it is easily trainable. Previous studies have demonstrated that factorizing the PPMI random walk transition matrix using SVD achieves better performance on downstream tasks compared to Skip-Gram derived models [35]. Referring to these studies, we state a hypothesis that our multilayer network embedding approach has similar computational advantages over other Skip-Gram-like network embedding models for multilayer graphs [21, 22, 24].

### Integrated omics network (ION) inference

**Fig. 4:**
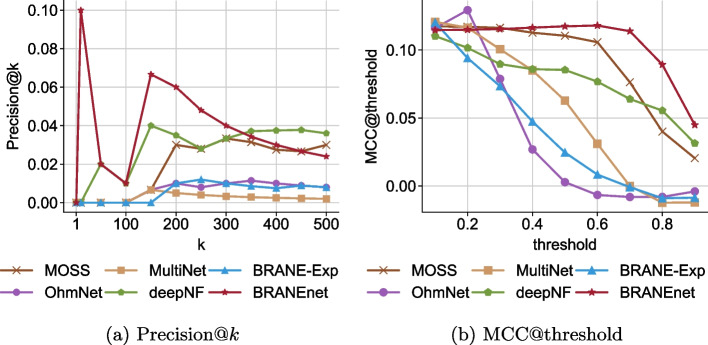
**a** Precision@*k* for top 500 edges compared to baseline methods. The *x*-axis represents top *k* edges and *y*-axis represents precision@*k* respectively. **b** MCC@threshold compared to baseline methods. The *x*-axis and *y*-axis represent threshold of $\delta$ and MCC@threshold, respectively

We infer an ION using the embeddings learned by BRANEnet. To validate the performance of ION inference, we reconstruct the gold-standard (GS) using the learned embeddings. The performance is measured by computing Precision@*k* and MCC (Mathews Correlation Coefficient). We choose to study the top 500 edges of the inferred ION. We compare the performance of our model to the performance of the baseline methods used. The results are shown in Fig. 4. The results of Precision@*k* show that BRANEnet scores, up to top 320 edges, are higher than deepNF. BRANEnet outperforms MOSS, OhmNet, MultiNet, deepNF, and BRANE-Exp (Fig. 4a). The results of MCC@threshold show that BRANEnet’s performance for different thresholds ($\theta$) is higher than OhmNet, MultiNet, deepNF, MOSS and BRANE-Exp. As shown in Fig. 4b, the MCC of BRANEnet was gradually improved with increasing threshold, and began to drop quite sharply at 0.6. For Precision@*k* metrics, deepNF is the second best performing model whereas for MCC@threshold, MOSS is the second best performing method. Moreover, we have also observed that, to obtain a well-trained model, methods like deepNF require large training data involving tuning of numerous parameters. This may cause overfitting/underfitting problems. The best performance of such baseline models can be achieved by extensive hyper-parameter tuning. As an advantage, BRANEnet comes with very few parameters. In the case of MOSS, the method is different from BRANEnet, pertaining to computation of random walk based PPMI matrix. This difference could make BRANEnet perform better than MOSS.

**Fig. 5:**
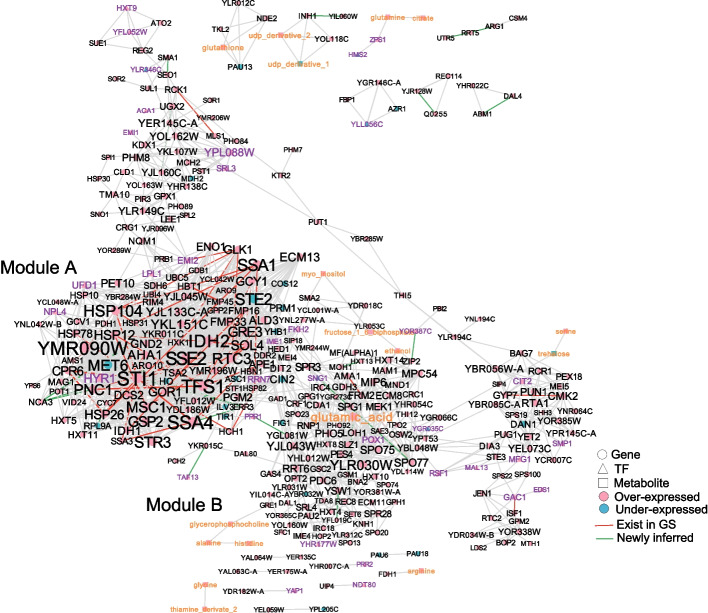
ION visualization for yeast during time-dependent heat stress inferred using BRANEnet. Node color, node shape and edge color represent the information shown in the legend. The label size of each node is proportional to the its degree in the inferred network

The network inferred by BRANEnet with $\theta = 0.7$ is shown in Fig. 5. Node colour represents over- (pink) and under- (blue) expressed bio-molecules. Node shape and label color represent genes (circle, black), TFs (triangle, purple), and metabolites (square, orange). Edges existing in GS are given in red whereas newly inferred edges are given in green. Edge width is represented by the similarity scores, while the node label size is proportional to its degree. Using the inferred ION, we narrow down the search space from all differentially expressed bio-molecules and identify potential biomarkers in heat stress response. We rank nodes based on their degree. Table 1 shows the obtained 21 bio-molecules that could be potential biomarkers in heat stress response. We have investigated the participation of these genes during heat-shock response in published literature. Using the BRANEnet integrated tool, we are able to recover information from 11 different heat shock response studies. The references of these articles are given in Table 1. We have also validated our results by comparing to another study of heat shock response [51]. We could find the potential bio-markers in the heat stress responsive gene clusters that were identified in this study.

**Table 1:** ION based identification of potential bio-markers

Name	$\uparrow /\downarrow$	${\mathcal {D}}$	Function	References	BRANEnet modules
STI1	$\uparrow$	40	Hsp90 cochaperone	[56–62]	A
SSA4	$\uparrow$	39	Heat shock protein	[58, 60–65]	A
TFS1	$\uparrow$	46	Inhibitor of carboxy-peptidase Y, Ras GAP	[56–58, 60–62, 64]	A
YMR090W	$\uparrow$	50	Unknown function	[56, 58, 61]	A
SSE2	$\uparrow$	36	Hsp110 family member	[56–58, 60–62, 64, 65]	A
IDH2	$\uparrow$	37	Oxidative decarboxy-lation of isocitrate	[57, 58, 61, 62]	A
SSA1	$\uparrow$	40	ATPase	[57–59, 61, 62, 64]	A
STE2	$\downarrow$	36	Receptor for $\alpha$-factor pheromone	[64]	A
HSP104	$\uparrow$	33	Disaggregase	[56–58, 60–62, 64, 65]	A
STI1	$\uparrow$	41	Hsp90 cochaperone	[56–62]	A
MET6	$\downarrow$	31	Cobalamin-independent methionine synthase	[57, 61]	A
STR3	$\uparrow$	30	Peroxisomal cysta-thionine beta-lyase	[57, 61, 64]	A
RTC3	$\uparrow$	28	Unknown function	[56–58, 61, 64]	A
MSC1	$\uparrow$	27	Unknown function	[56–58, 60–62, 64, 65]	A
PNC1	$\uparrow$	27	Nicotinamidase acid	[57, 58, 61, 62, 64, 65]	A
GSP2	$\uparrow$	30	GTP binding protein	[57, 58]	A
GRE3	$\uparrow$	31	Aldose reductase	[57, 60–62, 64, 66]	A
YLR030W	$\uparrow$	31	Unknown function	–	B
SOL4	$\uparrow$	32	6-phospho-gluconolactonase	[56–58, 61, 64]	A
HSP12	$\uparrow$	28	Heat shock protein	[56–58, 60–62, 64, 65]	A
IDH1	$\uparrow$	25	Oxidative decarboxy-lation of isocitrate	[57, 58]	A

The table provides the names, over- ($\uparrow$) or under- ($\downarrow$) expressed, node degree in ION (${\mathcal {D}}$), function, cross references, and BRANEnet module information comparison with external studies of potential bio-markers during heat stress response in yeast

### Biologically meaningful modules

**Fig. 6:**
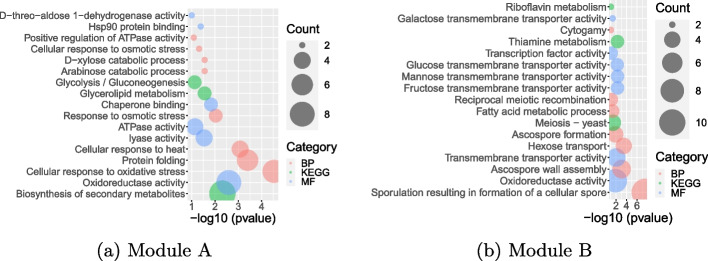
Functional enrichment of modules **A** and **B**. The *y*-axis represents the list of significantly enriched terms, while the *x*-axis shows their significance value ($-\log 10$(*p* value)). Different colors of circles indicate types of functional annotations: biological process (BP) in pink, molecular function (MF) in blue, and KEGG pathway in green. The size of circle represents the number of differentially expressed genes/TFs

To identify modules from the inferred ION, we perform community detection using the Clauset–Newman–Moore greedy modularity maximization algorithm [52]. We select modules with size more than 10 nodes. We have obtained 6 modules. To know if the obtained modules are biologically meaningful, we perform functional enrichment analysis on the two largest modules. We select the terms with *p* value lower than 0.05. Their enrichment results are shown in Fig. 6. We can clearly see that module A is enriched with catabolic processes including HSP90 and chaperone binding activity related terms while module B is enriched with transport and sporulation. The terms enriched in clusters A and B have been discussed over years in yeast heat-shock response studies [51, 53, 54].

## Parameter sensitivity analysis

**Fig. 7:**
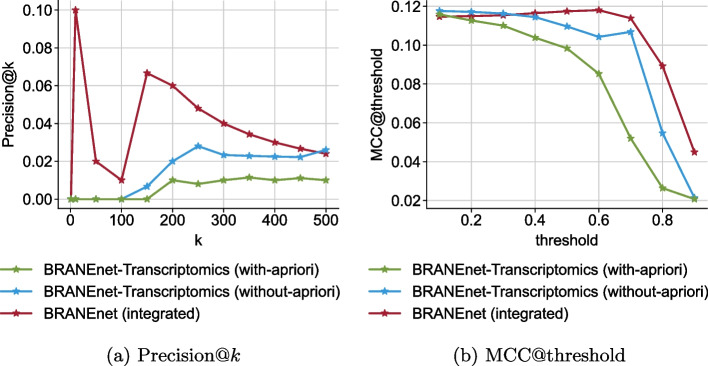
**a** Precision@*k* for top 500 edges. The *x*-axis represents top *k* edges and *y*-axis represents precision@*k* respectively. **b** MCC@threshold. The *x*-axis and *y*-axis represent threshold of $\delta$ and MCC@threshold, respectively

**Fig. 8:**
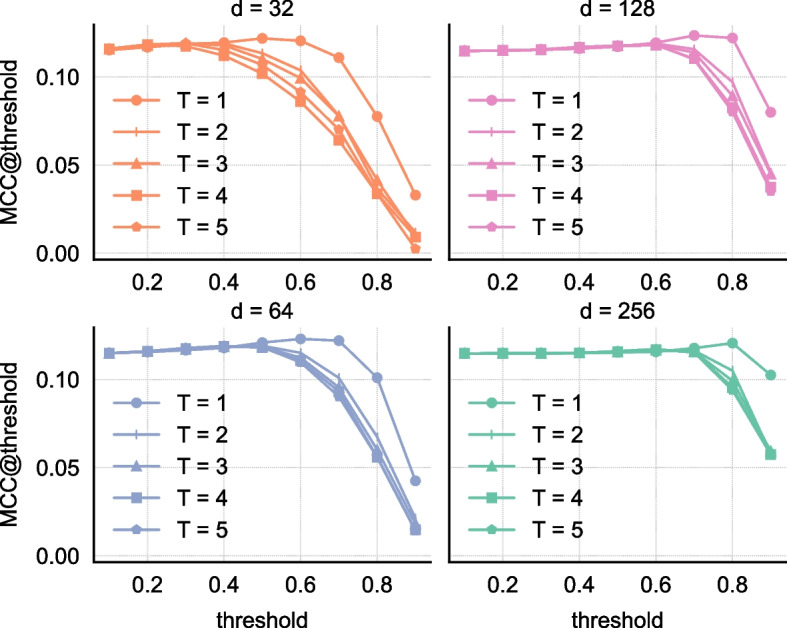
Parameter sensitivity analysis for ION inference. Node embeddings are computed using $T \in \{1,2,3,4,5\}$ and $d \in \{32,64,128,256\}$. The performance is measured by computing MCC for different values of $\theta$. The *x*-axis represents the MCC score at threshold ($\theta$) given in the *y*-axis

To examine the added value of integration, we have learned node embeddings by considering only one layer of information, i.e. transcriptomics. First, we consider only gene expression data and learn node embeddings. Secondly, we add the a priori knowledge to the transcriptomics data and learn node features. We compare the ION performance of using only one layer of information with the integrated embeddings acquired from multiple layers. Figure 7 shows that ION reconstruction is improved with the integration. We then investigated the robustness in the performance of BRANEnet with learned integrated embeddings. We used grid-search to assess the uncertainty in the model outputs that is attributed to different values of the window size *T* and dimension *d*. We choose $T \in \{1,2,3,4,5\}$, $d \in \{32,64,128,256\}$, and perform TF-target prediction and ION inference. The results for TF-target prediction are shown in Table 2. The mean AUPR in the given table for *average* and *weighted L2* is $83.8\%$ and $96.1\%$, respectively, with standard deviation of 2 percent. On the other hand, the results for ION inference are shown in Fig. 8. The performance is measured by MCC at different thresholds. From Fig. 8, the optimal threshold for $\theta$ is between 0.6 and 0.8. We also see that the performance of MCC is increased with respect to *d* and slightly decreased with *T*. From the parameter sensitivity analysis for both tasks, we see that our model has lower variance in the results with respect to different parameter settings. Therefore for the new datasets, we recommend users to consider the default parameter settings ($T=3$ and $d=128$).

**Table 2:** Parameter sensitivity analysis for TF-target prediction

	d = 32	d =64	d =128	d =256
Average				
T =1	0.700	0.880	0.880	0.870
T = 2	0.790	0.820	0.850	0.860
T =3	0.830	0.850	0.870	0.870
T =4	0.780	0.810	0.850	0.860
T =5	0.820	0.840	0.860	0.870
Weighted L2				
T =1	0.980	0.982	0.983	0.983
T =2	0.916	0.945	0.966	0.968
T =3	0.956	0.967	0.979	0.979
T =4	0.852	0.938	0.966	0.968
T= 5	0.952	0.968	0.981	0.981

Node embeddings are computed using $T \in \{1,2,3,4,5\}$ and $d \in \{32,64,128,256\}$. The performance is measured by computing AUPR score for *average* and *weighted L2* coordinate-wise operations

## Conclusions

The recent wide application of high-throughput experimental techniques has provided complex high-dimensional heterogeneous data. In turn, the wide availability of these omics data has driven the need to develop methods that can efficiently analyse and integrate this data. We have presented an integrative analysis of multilayer heterogeneous networks for learning low-dimensional features for bio-molecules. BRANEnet relies on a GRL technique to learn embeddings that can capture relevant features from complex networks built from single omics data. Considering the biological context and need for omics integration, methods like BRANEnet enable us to study the cross-talk between genes, transcription factors, and metabolites at transcriptional and metabolic levels of regulation.

Besides, we have compared the performance of our approach for various downstream tasks with existing network integration methods. To summarize the empirical analysis, the performance of BRANEnet is especially appealing mainly because of four reasons. First, our approach integrates experimental data with biological a priori knowledge which facilitates the inference of inter-omics relationships. Second, it can generate meaningful embeddings by preserving the inter- and intra-omics interactions. Third, its objective function is independent of the downstream tasks, thereby it is adaptable to various omics data inference tasks. And fourth, it has a low number of parameters to tune. For the predicted bio-markers, experimental as well as in silico investigation can help in identifying their functions and biological significance [55]. As a future work, we intend to test BRANEnet on multiple omics datasets including epigenetics, proteomics, and metagenomics. We would also like to explore promising directions of multilayer network embedding that can support fast approximations of higher-order random walks transition matrices. BRANEnet is a versatile tool that combines data-driven information and biological a priori knowledge and provides an effective and scalable network integration framework with diverse downstream integrative omics applications. We consider BRANEnet to be a valuable method of biological network integration for experimental follow-up, as well as a baseline candidate for further development of the graph-based multi-omics data integration field.

## Supplementary Information

**Additional file 1.** The list of all DE bio-molecules with their FC and SGD annotation.

## Data Availability

All data generated or analyzed during this study are included in this published article, its supplementary file and the GitHub repository. BRANEnet is freely available as a Jupyter notebook present at https://github.com/Surabhivj/BRANEnet. Zenodo: https://doi.org/10.5281/zenodo.7007534.
